# Supplementary material for: Observational learning of fear in real time procedure
Source: Sci Rep. 2020 Oct 12;10:16960. doi: 10.1038/s41598-020-74113-w (PMC7550349; doi:10.1038/s41598-020-74113-w)
Supplement: Supplementary file 1 — Supplementary Information. [file 41598_2020_74113_MOESM1_ESM.docx]

Supplementary material for:

**Observational Learning of Fear in Real Time Procedure**

Michał Szczepanik, Anna M. Kaźmierowska, Jarosław M. Michałowski, Marek Wypych, Andreas Olsson, and Ewelina Knapska

# Supplementary Methods

## Instructions given to the participants (translation)

The demonstrator’s role explanation: *You are the demonstrator. It means that you will be asked to do the task and your performance will be observed by your friend. In the task you will be shown colored figures but only one of them – the blue/yellow one* (depending on the experiment version) *– will be paired with an electric shock administered to the forearm in 50 percent of appearances. It will be very unpleasant but not painful. Please signal the shock occurrences clearly, so that your friend knows that they cause your discomfort. Besides the natural twitch of the forearm, please also try to react with a facial grimace of discomfort, e.g. squint your eyes and frown your forehead. It is very important that you react distinctly only in response to the shock administration – please try not to react to any other stimuli.*

The observer’s role explanation: *You are the observer. It means that you will be asked to watch your friend performing the task. During the task both of you will see colored figures presented on his computer screen and hear short, loud sounds. The sounds will not be associated with the task though, so please try to ignore them. Your friend will receive electric shocks several times during the task. The shocks will be very unpleasant but not painful and they will induce certain reaction in your friend. Please focus on your friend performing the task. Later on, you will be asked to do the same task yourself.*

## Note on sample size

In a recent SCR study examining effects of social group biases on observational fear conditioning^1^ , the effect size in the related samples t-test (CS+ vs CS- during the early stage of the direct test) reported for the subgroup of observers most comparable with the current study (racial and social in-groups with the demonstrator) and calculated based on supplementary data was d = 0.82. By convention, such effect is considered as large. Assuming d = 0.82, a sample size of just 14 subjects is sufficient to achieve 80 % power, as calculated using G*Power.

## Technical considerations

Electrical stimulation was delivered using Biopac STM100C and STMISOC stimulator modules, driven by a National Instruments USB-6001 analog output card. Two Ag/AgCl laminated, carbon composition contact electrodes placed 3.5 cm apart (measured between centers) and filled with salt free electrode gel were used for that purpose.

Using an HD-SDI security camera with an HDMI converter has an advantage of low latency and high robustness: SDI signal can be transmitted over long distances using a concentric cable, such setup is easy to connect and does not involve a PC for video processing. Using an action camera with direct HDMI output can be equally convenient, but unamplified HDMI cables are usually limited to 15 meters, which should be taken into consideration. In both cases, demonstrator’s screen brightness and camera white balance should be carefully adjusted to ensure accurate representation of conditioned stimuli.

## Physiological recordings

Electrophysiological measurements were collected only from the observers. A Biopac MP-160 acquisition system and AcqKnowledge 4.0 software (Biopac Systems Inc., Goleta, CA, USA) were used to collect the data. Skin conductance was recorded using a Biopac EDA100C amplifier (gain: 5μS/V, 10 Hz low-pass hardware filter) and sampled at 2 kHz. Two 6 mm Ag-AgCl electrodes were attached to the distal phalanges of the second and third digits of the participant’s left hand. Startle response was recorded using a Biopac EMG100C amplifier (gain: 2000, 1 Hz high-pass, 500 Hz low-pass hardware filter) and sampled at 2 kHz. Two Ag-AgCl shielded electrodes were attached under participant’s right eye, on the orbicularis oculi muscle (one electrode in line with the pupil in forward gaze, the second 1-2 cm laterally). The ground electrode (identical, except non-shielded) was placed on the participant’s forehead, directly under the hairline.

# Supplementary data and analyses

## Questionnaire results

Polish adaptations of State-Trait Anxiety Index (STAI) and The Basic Empathy Scale in Adults (BES-A) were used. STAI consists of two 20-item subscales: STAI-State and STAI-Trait. The BES-a is a 20-item scale which can be divided into three subscales: emotional contagion, cognitive empathy and emotional disconnection, here treated separately. At the beginning of the experiment (after the roles were assigned, but before role-specific instructions were given to participants) the participants completed the STAI-state. After OFL, the demonstrators completed the STAI-state for the second time. At the end of the experiment (i.e. after DE) the observers filled in the STAI-state (for the second time), as well as STAI-trait and BES-A. Summary statistics are presented in the Supplementary Table 1.

Supplementary Table 1: Summary statistics of the questionnaire results. Me - median, IQR - interquartile range. For STAI state, before and after refer to the beginning of the experiment and either end of OFL (demonstrators) or end of DE (observers).

| **Measure** | **Min** | **Max** | **Me** | **IQR** |
| --- | --- | --- | --- | --- |
| *demonstrators* | | | | |
| STAI state (before) | 20 | 55 | 34 | 8 |
| STAI state (after) | 21 | 53 | 30 | 8.5 |
| *observers* | | | | |
| STAI state (before) | 23 | 54 | 34 | 7.5 |
| STAI state (after) | 22 | 49 | 31 | 8.5 |
| STAI trait | 29 | 63 | 43 | 10.25 |
| BES emotional contagion | 7 | 28 | 20 | 6.75 |
| BES cognitive empathy | 17 | 39 | 32 | 3.75 |
| BES emotional disconnection | 6 | 29 | 14 | 5.75 |

The scores were screened for the presence of outliers, defined as being 3 standard deviation above or below group mean. Two such results were found: one participant had a score of 17 points in BES emotional contagion subscale (3.31 SD below mean) and another participant had 29 points in BES emotional disconnection subscale (3.00 SD above mean). Additionally, STAI-trait and BES-A questionnaires from one subject are missing. No data exclusions were performed on that basis.

Results of the STAI-state questionnaire collected before and after the fear learning procedure were compared using a paired t-test revealing a significant difference for both observers, t(34) = 2.57, p = 0.01, and demonstrators, t(34) = 2.50, p = 0.02, with higher values at the beginning of the experiment (mean of the differences: 2.26 and 2.74 respectively, see Supplementary Figure 1). This difference can be explained by the fact that participants likely showed elevated anxiety at the beginning of the experiment and that the second measurement took place after completion of tasks, when the participants knew they would not experience any more aversive stimuli.

Results from STAI and BES-A subscales were compared between the contingency-aware and not-aware observers using non-parametric Kruskal-Wallis rank sum tests, which showed no significant differences between the two groups (highest χ^2^ = 0.90 obtained for BES emotional disconnection, corresponding p = 0.34, uncorrected for multiple comparisons).

## Associations between questionnaire results and conditioned responding

The scores obtained from STAI and BES-A questionnaires as well as ratings given by the observers were correlated with the observers’ physiological measures of responding: unconditioned response (obs US > noUS), SCR conditioned response (direct CS+ > CS-) and FPS conditioned response (direct CS+ > ITI). Correlation matrix was calculated using Psych package in R ([https://CRAN.R-project.org/package=psych](https://cran.r-project.org/package=psych), version 2.0.7) and is shown in Supplementary Table 2. Notably, only modest and not statistically significant correlations were found between the psychometric and physiological variables.


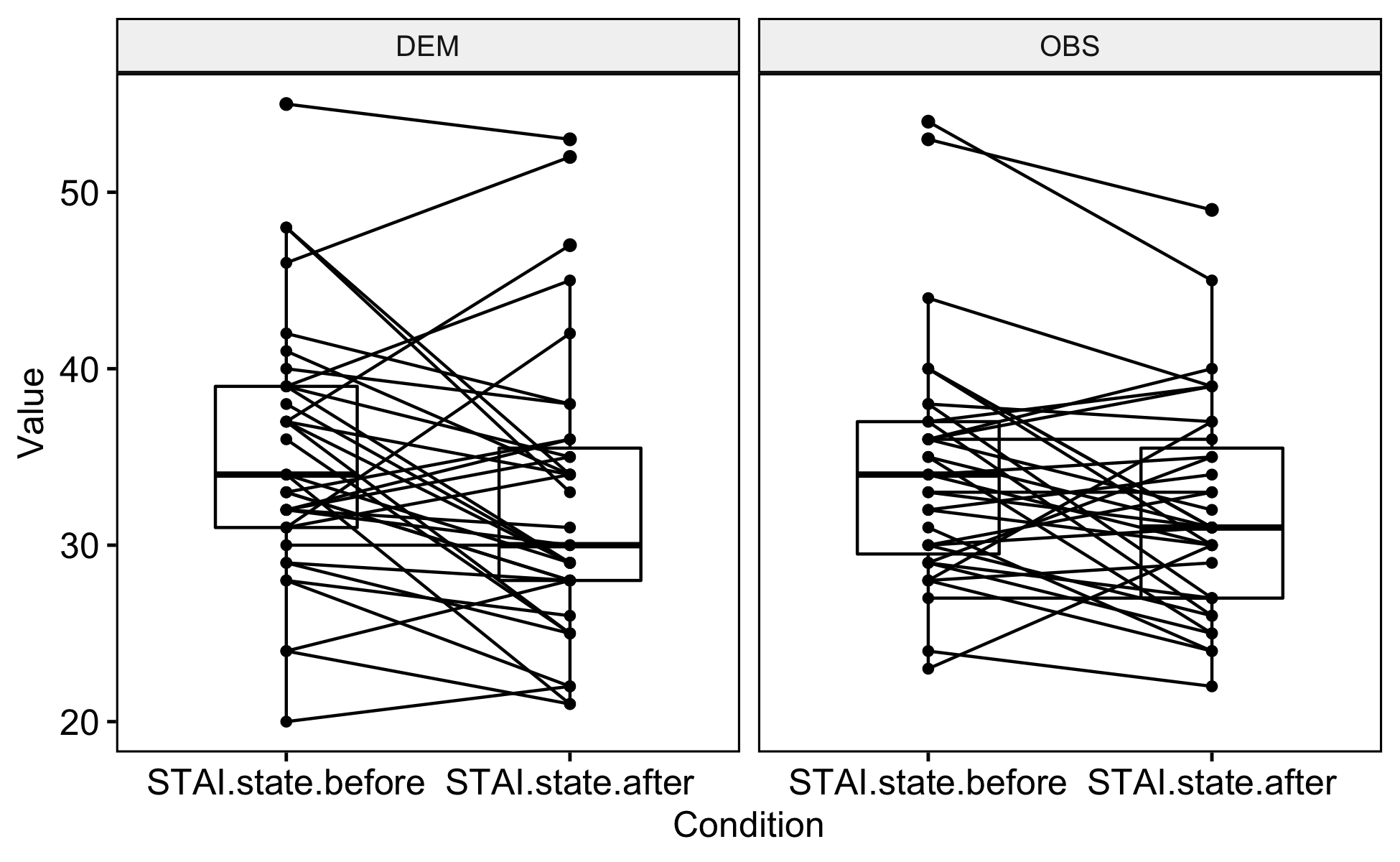


Supplementary figure 1: Comparison of STAI-state scores of the observers before starting the experiment and after its completion. Results at the beginning were higher than at the end for both demonstrators (t=2.50, df=34, p=0.02, mean of the differences) and observers (t = 2.57, df = 34, p = 0.01; mean of the differences: 2.26). Plot made using ggpubr R package ([https://CRAN.R-project.org/package=ggpubr](https://cran.r-project.org/package=ggpubr), version 0.4.0).

Supplementary table 2: Pearson correlations between psychometric and physiological measures for observers. STAI and BES indicate respective questionnaires, while R prefixes the responses of the observer about the observation task. *: p < .05; **: p < .01; ***: p < .001 (uncorrected for multiple comparisons)

|  | **STAI state before** | **STAI state after** | **STAI trait** | **BES emotional contagion** | **BES cognitive empathy** | **BES emotionaldisconn.** | **R**  **discomfort** | **R reaction strength** | **R reaction natural.** | **R empathy felt** | **R**  **identify** | **SCR**  **diff. US** | **SCR**  **diff. CS** | **FPS**  **diff. CS** |
| --- | --- | --- | --- | --- | --- | --- | --- | --- | --- | --- | --- | --- | --- | --- |
| **STAI-state before** |  |  |  |  |  |  |  |  |  |  |  |  |  |  |
| **STAI-state after** | .68*** |  |  |  |  |  |  |  |  |  |  |  |  |  |
| **STAI-trait** | .41 | .37 |  |  |  |  |  |  |  |  |  |  |  |  |
| **BES em. cont** | .28 | .40 | .36 |  |  |  |  |  |  |  |  |  |  |  |
| **BES c. empathy** | .05 | .12 | .04 | .47** |  |  |  |  |  |  |  |  |  |  |
| **BES e. disconn.** | .05 | -0.22 | -0.11 | -0.68*** | -0.54*** |  |  |  |  |  |  |  |  |  |
| **R discomfort** | -0.10 | -0.05 | .27 | .14 | .07 | -0.21 |  |  |  |  |  |  |  |  |
| **R strength** | -0.27 | -0.08 | -0.07 | .00 | .08 | -0.37 | .36 |  |  |  |  |  |  |  |
| **R naturalness** | -0.19 | -0.06 | -0.00 | .45** | .22 | -0.56*** | .26 | .37 |  |  |  |  |  |  |
| **R empathy** | -0.06 | .16 | .15 | .55*** | .29 | -0.63*** | .50** | .49** | .44** |  |  |  |  |  |
| **R identify** | -0.26 | -0.07 | -0.30 | .18 | .48** | -0.46** | .11 | .23 | .27 | .52** |  |  |  |  |
| **SCR diff. us** | .21 | .26 | -0.01 | .19 | .01 | -0.15 | -0.32 | .00 | .04 | -0.10 | -0.08 |  |  |  |
| **SCR diff. CS** | .01 | .03 | .10 | .18 | .19 | -0.27 | -0.27 | -0.33 | -0.13 | -0.11 | .25 | .07 |  |  |
| **FPS diff. cs** | .04 | -0.00 | -0.08 | -0.04 | .23 | -0.19 | .09 | .25 | -0.10 | .13 | .26 | -0.27 | .25 |  |

## Analysis of early trials in DE

To test whether the effects reported for Direct Expression are stable with respect to averaging over the entire task versus its first half (6 CS+ and 6 CS-, further referred to as early trials), we conducted additional ANOVAs using afex package in R (<https://cran.r-project.org/package=afex>, version 0.27.2). Greenhouse-Geisser corrections for sphericity were applied as needed. Post-hoc tests with Bonferroni correction were calculated using package emmeans (<https://cran.r-project.org/package=emmeans>, version 1.4.8).

In SCR, when averaging over early trials, we observed a significant stimulus:contingency interaction, $F(1,30)=9.41$, $\eta_{G}^{2}=.100$, $p=.005$. We additionally observed a main effect of contingency, $F(1,30)=5.86$, $\eta_{G}^{2}=.112$, $p=.022$ (which is not significant when all trials are considered). The CS+ > CS- contrast (averaged over the levels of contingency) remained not significant, $t(30)=1.41, p =.17$. When taking contingency awareness into account, there was a significant CS+ > CS- difference in contingency aware participants $t(30)=2.987, p=.022$, as well as aware > unaware difference in responding to the CS+, $t(55.3)=3.771, p=.002$.

In FPS, when averaging over early trials, we observed a main effect of stimulus, $F(1.95,58.49)=9.92$, $\eta_{G}^{2}=.181$, $p<.001$, however the stimulus:contingency interaction (trend-level for all trials) was not significant, $F(1.95, 58.49)=1.99$, $\eta_{G}^{2}=.042$, $p=.147$. The CS+ > ITI and CS- > ITI contrasts (averaged over the levels of contingency) remained significant, $t(60)=4.296, p < .001$ and $t(60)=3.166, p =.007$ respectively; the CS+>CS- contrast remained not significant, $t(60)=1.130, p =.789$. When taking contingency awareness into account, there was (only) a significant CS+ > ITI difference in contingency aware participants, $t(60)=4.070, p = .001$, while the CS- > ITI difference in contingency aware participants (trend-level for all trials) was not significant, $t(60)=2.550, p = .12$.

**Supplementary References**

1. [Golkar, A. & Olsson, A. The interplay of social group biases in social threat learning. *Sci. Rep.* **7**, 7685 (2017).](http://paperpile.com/b/YcHn6Z/sMAt)
